# Novel Synthetic Monoketone Transmute Radiation-Triggered NFκB-Dependent TNFα Cross-Signaling Feedback Maintained NFκB and Favors Neuroblastoma Regression

**DOI:** 10.1371/journal.pone.0072464

**Published:** 2013-08-14

**Authors:** Sheeja Aravindan, Mohan Natarajan, Vibhudutta Awasthi, Terence S. Herman, Natarajan Aravindan

**Affiliations:** 1 Department of Radiation Oncology, University of Oklahoma Health Sciences Center, Oklahoma City, Oklahoma, United States of America; 2 Center for Advanced Study, Annamalai University, Parangipettai, Tamil Nadu, India; 3 Stephenson Cancer Center, Oklahoma City, Oklahoma, United States of America; 4 Department of Pathology, University of Texas Health Sciences Center at San Antonio, San Antonio, Texas, United States of America; 5 Department of Pharmaceutical Sciences, University of Oklahoma Health Sciences Center, Oklahoma City, Oklahoma, United States of America; 6 Department of Pathology, University of Oklahoma Health Sciences Center, Oklahoma City, Oklahoma, United States of America; 7 Department of Anesthesiology, University of Oklahoma Health Sciences Center, Oklahoma City, Oklahoma, United States of America; Vanderbilt University, United States of America

## Abstract

Recently, we demonstrated that radiation (IR) instigates the occurrence of a NFκB-TNFα feedback cycle which sustains persistent NFκB activation in neuroblastoma (NB) cells and favors survival advantage and clonal expansion. Further, we reported that curcumin targets IR-induced survival signaling and NFκB dependent hTERT mediated clonal expansion in human NB cells. Herein, we investigated the efficacy of a novel synthetic monoketone, EF24, a curcumin analog in inhibiting persistent NFκB activation by disrupting the IR-induced NFκB-TNFα-NFκB feedback signaling in NB and subsequent mitigation of survival advantage and clonal expansion. EF24 profoundly suppressed the IR-induced NFκB-DNA binding activity/promoter activation and, maintained the NFκB repression by deterring NFκB-dependent TNFα transactivation/intercellular secretion in genetically varied human NB (SH-SY5Y, IMR-32, SK–PN–DW, MC-IXC and SK–N-MC) cell types. Further, EF24 completely suppressed IR-induced NFκB-TNFα cross-signaling dependent transactivation/translation of pro-survival *IAP1, IAP2* and *Survivin* and subsequent cell survival. In corroboration, EF24 treatment maximally blocked IR-induced NFκB dependent *hTERT* transactivation/promoter activation, telomerase activation and consequent clonal expansion. EF24 displayed significant regulation of IR-induced feedback dependent NFκB and NFκB mediated survival signaling and complete regression of NB xenograft. Together, the results demonstrate for the first time that, novel synthetic monoketone EF24 potentiates radiotherapy and mitigates NB progression by selectively targeting IR-triggered NFκB-dependent TNFα-NFκB cross-signaling maintained NFκB mediated survival advantage and clonal expansion.

## Introduction

In United States, each year 650 children are diagnosed with neuroblastoma (NB) [1], an embryonal malignancy of sympathetic nervous system that is remarkable for its clinical heterogeneity [2]. Patients with the invasive/metastatic high-risk stage-IV NB showed refraction with all conventional therapeutic modalities and is associated with dismal prognosis [3]. High recurrence rate (20.2%) entailing substantial fractions of both local (17%-74% of patients) [4–8] and distant metastasis (46.8%) present considerable challenges for the clinical management of NB. With only 13 months since first diagnosis to recurrence, the survival ratio was 43% for local and 10% for systemic recurrences. Clinical and laboratory evidence suggests that several human cancers contain populations of rapidly proliferating clonogens that can have substantial impact on local control following chemoradiotherapy [9]. Tumor cell repopulation may arise from remnant cells of the original neoplasm that have escaped therapeutic intervention and later become visible at the original site. Radiotherapy (RT), now widely used for high-risk NB patients after chemotherapy, significantly improved the survival rate [10]. Patients, however, have been confronted with relapse and developed drug/radiation resistance, possibly through favoring alternative pathways. Therapeutic doses of radiation (IR) has been shown to activate various transcription factors including NFκB [11] and studies have suggested their influential role in tumorigenesis and progression [12]. Recently, we demonstrated the radiation triggered NFκB initiates TNFα cross signaling dependent maintenance of NFκB that in turn promotes survival advantage in both *in vitro* and *in vivo* NB models [13]. To that end, identifying ‘drug-deliverables’ that selectively disrupt IR-induced NFκB-TNFα feedback signaling and impedes NFκB maintenance could deter NFκB-dependent survival advantage and potentiate RT in NB cure.

NB exhibits a remarkable heterogeneity with respect to clinical behavior, ranging from spontaneous regression or differentiation with favorable outcome to a rapid progression with poor outcome, despite multimodal therapy. Recently, we dissected out that IR induced NFκB in human NB cells [14,15] is responsible for the induced *TERT* transcription, enhanced TA and subsequent clonal expansion [16]. In this context, clearly, there is a need to recognize new, effective and clinical-translation feasible drugs that selectively target radiation induced NFκB-dependent TERT to mitigate clonal expansion and NB progression. Concurrently, we have shown that curcumin (diferuloylmethane), a polyphenol, sensitizes NB cells to the apoptotic effects of radiation [14] and, further mitigates radiation-induced NFκB-dependent *TERT* transcription, TA and subsequent clonal expansion [16]. However, the full potential of curcumin has not been realized because of the relatively poor bioavailability in the clinical settings [17,18]. To that end, a synthetic analog of curcumin, EF24 (3,5-Bis(2-flurobenzylidene) piperidin-4-one), with better pharmacokinetic and improved physiochemical properties has been well tolerated in animal models [19,20]. EF24 has been shown to possess anti-tumorigenic [21–23] activity and has been demonstrated to directly inhibit IKKβ, a probable explanation for the improved therapeutic potency over curcumin [24]. We recently have shown that EF24 suppresses NFκB dependent inflammation in dendritic cells [25]. Considering the immense potential of EF24 as an anti-cancer agent, a parenteral formulation for EF24 will be greatly beneficial in preclinical and clinical trials. In this regard, investigating the efficacy of EF24 in disrupting the IR-induced molecular signal transduction (here in this case, NFκB-TNFα cross-signaling), inhibiting persistent activation of NFκB, and, reverting induced survival advantage, clonal expansion, NB dissemination to distant sites in response to IR will prove highly beneficial in achieving the desired therapeutic gain in treating NB. Accordingly, using both *in vitro* and *in vivo* NB model, we investigated whether EF24 could selectively target and inhibit RT-induced NFκB-TNFα-NFκB cross signaling-dependent persistent activation of NFκB and thereby offer a comprehensive and complete prevention of NFκB-mediated survival advantage, clonal expansion and tumor relapse.

## Materials and Methods

### Ethics Statement

All animal experiments conformed to American Physiological Society standards for animal care and were carried out in accordance with guidelines laid down by the National Research Council and were approved by University of Oklahoma Health Sciences Center – Institutional Animal Care and Use Committee.

### Cell Culture

Human SH-SY5Y, IMR-32, SK–PN–DW, MC-IXC and SK–N-MC cells were obtained from ATCC (Manassas, VA). Culture and maintenance of SH-SY5Y, IMR-32 and SK–N-MC cells were performed as described earlier [26]. SK–PN–DW and MC-IXC cells were maintained in DMEM medium (Mediatech Inc., Herndon, VA) supplemented with 5000 I.U/ml penicillin/5000 µg/ml streptomycin and 10% FBS. For all the experiments, the cells were serum-starved by incubating in 2% serum for at least 16h, unless otherwise specified.

### Irradiation, EF24 treatment and Inhibition studies

For IR experiments, cells were exposed to 2Gy using Gamma Cell 40 Exactor (Nordion International Inc, Ontario, Canada) at a dose rate of 0.81Gy/min and incubated at 37° C for additional 1, 3, 6, 24, 48 and 72h. Synthesized EF24 [25,27] was dissolved in DMSO to a stock-concentration of 100mM and was diluted in plain media to a working-concentration of 50μM. For EF24 alone treatment, cells were treated with 50, 100, 200nM or 1μM EF24 and allowed to incubate for 24h. To determine the effect of EF24 on IR-induced modulations, cells treated with EF24 (3h) were then exposed to IR (2Gy). For TNFα inhibition studies, cells were treated with 100ng/ml TNFR1 antibody (Santa Cruz biotech, Santa Cruz, CA) and then exposed to IR as described earlier [26].

### Plasmid preparation, DNA Transfection and Luciferase reporter assay

Transient transfection of NFκB p65/p50 subunits was carried out as described in our earlier studies [13,28]. NFκB inhibition was achieved using siRNAs targeting RelA (Qiagen) as described earlier [13]. EF24 treatment associated regulation of IR-induced NFκB and TERT promoter activation were investigated using luciferase reporter assay [16,26].

### Electrophoretic Mobility Shift Assay

Nuclear protein extraction and electrophoretic mobility shift and specificity assays were performed as described in our earlier studies [13–15].

### QPCR

The effect of EF24 on IR-induced NFκB-dependent regulation of *TNFα* and *hTERT* mRNA expression and persistent activation of NFκB dependent transcriptional response of *cIAP1*, *cIAP2* and *Survivin* were analyzed by real-time QPCR as described earlier [13] [14]. We used β-actin as a positive control, and a negative control without template RNA was also included. Each experiment was carried out in triplicate, and the ^ΔΔ^ *C*t values were calculated by normalizing the gene expression levels to β-actin, and the relative expression level was expressed as a fold change over mock-irradiated (untreated) control.

### ELISA

TNFα ELISA in concentrated medium was performed as described in our earlier studies [13,29].

### Immunoblotting

Total protein extraction and immunoblotting were performed as described in our earlier studies [13,30]. For this study, the protein transferred membranes were incubated with rabbit polyclonal anti-cIAP1, cIAP2, Survivin or mouse polyclonal anti-pIκBα antibody (Santa Cruz).

### Cell survival by MTT and clonogenic assay

Cell survival was analyzed using MTT and clonogenic assays as described in our previous studies [13,14].

### Telomerase Activity Assay

Alterations in telomerase activity was examined using TRAP assay as described our earlier studies [16].

### In vivo Xenograft Experiments and FDG-PET-CT imaging

Seven weeks old athymic NCr-*nu/nu* nude mice (NCI, Frederick, MD) received SH-SY5Y (*s.c.* 5x10^6^) cells suspended in Matrigel (BD Biosciences) into their right flank. Xenografts were selectively exposed to 2Gy-FIR (2Gy/day, to a total dose (TD) of 20Gy), 5Gy-FIR (5Gy/day for 15 days, TD 75Gy) or 10Gy-FIR (10Gy/day for 7 days, TD 70Gy) using a specially designed cerrobend shield. For EF24 alone experiments, animals received daily dose of 50, 100 or 200mg/Kg EF24 (*i.p.*). To study the radiosensitization potential of EF24, xenografts were exposed to 2Gy-FIR, 5Gy-FIR or 10Gy-FIR in conjunction with daily-dose of either intra-tumoral (for 2Gy-FIR) or intra peritoneal (for 5 and 10Gy-FIR) EF24 (200μg/Kg) 3h prior to IR. Tumor progression/regression was detected with Positron Emission Tomography (PET) using F-18- fluorodeoxyglucose (FDG). For this, overnight-fasted animals were injected (*i.v.*, 0.3 mCi) with FDG for 1h and imaged using X-PET (Gamma Medica-Ideas, CA). An x-ray CT image was also acquired to establish anatomical landmarks. Acquired image data was reconstructed using filtered back projection. Tumor growth and regression was also estimated by comparing tumor volume measurements using calipers.

## Results

### EF24 inhibits IR-induced activation and maintenance of NFκB

**Figure 1:**
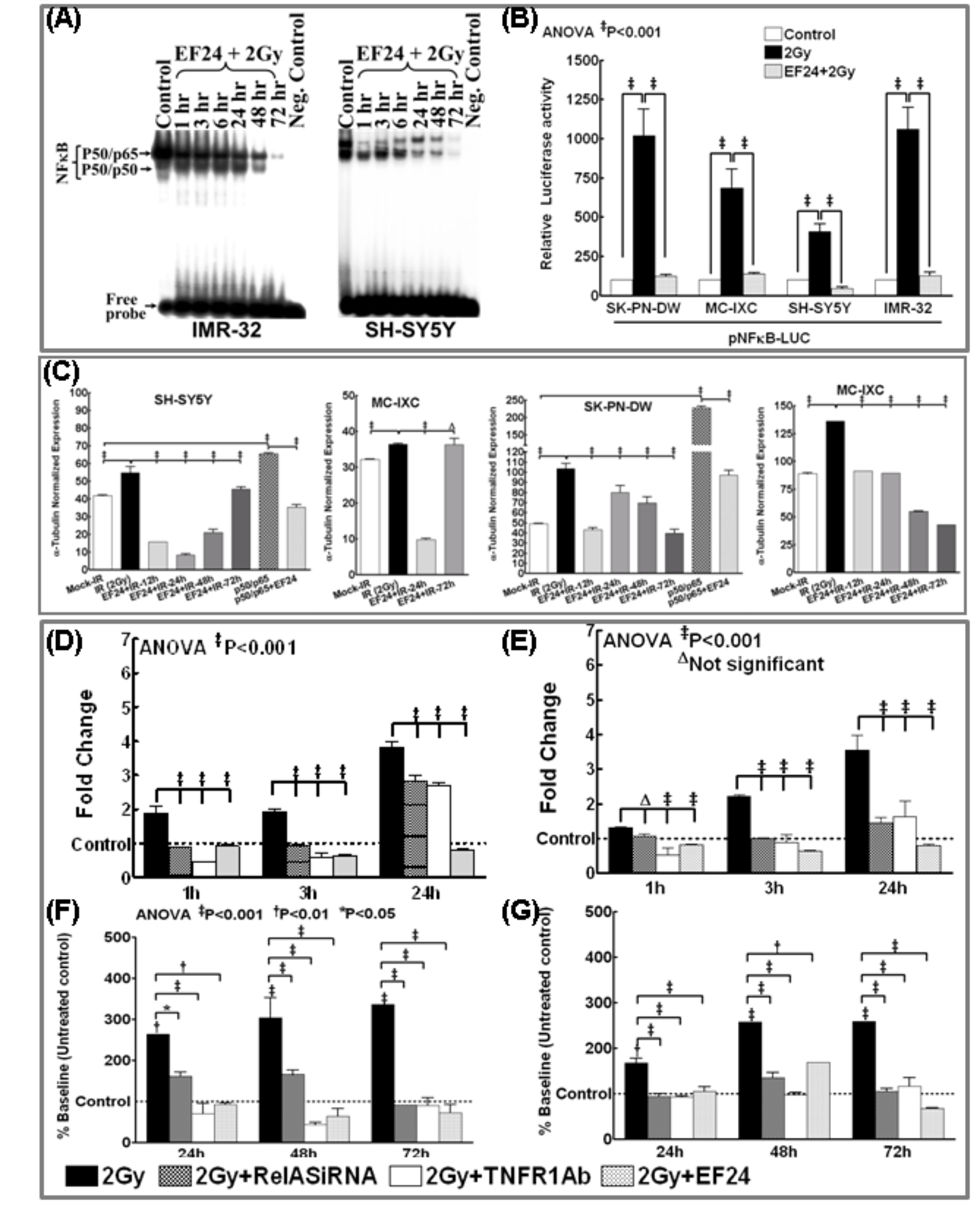
EF24 inhibits radiation-induced DNA binding activity and transcriptional activation of NFκB; transactivation and secretion of TNFα in human neuroblastoma cells. (**A**) NFκB DNA-binding activity in SH-5Y5Y and IMR-32 cells treated with EF24 (200nM) and exposed to 2Gy. The cells were incubated in a CO_2_/air incubator for additional 1, 3, 6, 24, 48 and 72h. The nuclear extracts were analyzed by EMSA using γ-^32^p[ATP] labeled NFκB-specific probe. Autorads were overexposed to capture the reduced activities that were equal or lower than mock-IR controls. (**B**) Luciferase reporter assay: SH-SY5Y, IMR-32, SK–PN–DW and MC-IXC cells transfected with pNFκB-Luc construct and either mock irradiated, exposed to 2Gy, or treated with EF24 and exposed to 2Gy were harvested at 24h post-IR and analyzed by luciferase assay. Data shown represent the mean and SD of three independent experiments. (**C**) Semi-quantitative densitometry of immunoblots using Quantity One 1D image analysis Version 4.6.5 (Biorad) showing α-tubulin intensity normalized expression of pIκBα in SH-SY5Y, IMR-32, SK–PN–DW and MC-IXC cells. Cells either mock-irradiated, exposed to 2Gy and harvested after 24h, treated with EF24 for 3h followed by 2Gy exposure and harvested after 1, 3, 12, 24, 48 and 72h, transfected with p50/p65 for 24h or transfected with p50/p65 for 24h and treated with EF24 for additional 24h. Groups were compared using Two-way ANOVA with Bonferroni’s Post-hoc correction. (**D** & **E**) Real time QPCR analysis showing *TNFα* mRNA expression in human neuroblastoma cells: (**D**) SH-SY5Y and (**E**) IMR-32 cells exposed to IR (2Gy) with or without EF24 treatment, transfected with RelA siRNA and exposed to 2Gy or treated with TNFR1 Ab and exposed to 2Gy and harvested after 1, 3 and 24h. The ΔΔ^ct^ values were calculated by normalizing the gene expression levels to internal housekeeping gene (*β-actin*), compared between groups, and the relative expression level was expressed as a fold change over mock-IR cells. (**F** & **G**) ELISA analysis showing intercellular TNFα levels in (**F**) SH-SY5Y and (**G**) IMR-32 cells either exposed to IR (2Gy) with or without EF24, transfected with RelA siRNA and exposed to IR or treated with TNFR1 Ab and exposed to 2Gy. Conditioned medium from the cells were recovered after 24, 48 or 72h, concentrated (9KD concentrators) and subjected to ELISA. Group-wise comparisons were made using ANOVA with Tukey’s post-hoc correction.

To determine the influence of EF24 in impeding IR-associated induction and maintenance of NFκB activity, SH-SY5Y and IMR-32 cells exposed to IR with/without EF24 (200nM for 3h) were examined after 1h through 72h. EF24 alone (10, 20, 50, 100, 200nM) treated cells showed a dose-dependent inhibition of NFκB DNA binding activity with a maximal inhibition at 200nM (data not shown). Recently, we showed that at least in human NB cells, IR persistently induced NFκB for up to 3 days in a NFκB-TNFα positive feedback-dependent manner [13]. In this study, treating NB cells with EF24 significantly suppressed this IR-induced NFκB activity. EF24-associated suppression of IR-induced NFκB was observed as early as 1h post-IR and remained consistent for up to 72h in both cell lines (Figure 1A). Together, these data suggests that EF24 not only attenuates the IR-triggered immediate early NFκB response, but potentially mitigates the NFκB-TNFα PFC-dependent NFκB maintenance in these cells.

Further to substantiate our findings, we investigated whether EF24 attenuate IR-induced NFκB promoter activation 24h post-IR which would validate whether EF24 disrupts the second signaling (NFκB-TNFα-NFκB) feedback dependent maintenance of NFκB. SH-SY5Y, IMR-32, SK–PN–DW and MC-IXC cells transfected with pNFκB-Luc plasmid construct that expresses the luciferase reporter gene in a NFκB-dependent manner exposed to IR with/without EF24 were subjected to luciferase reporter assay after 24h. Compared to mock-IR, 2Gy exposure induced a colossal (P<0.001) increase in luciferase activity, indicating that IR could specifically maintain NFκB functional transcription in all four cell lines investigated (Figure 1B). Conversely, EF24 pretreatment resulted in a complete (P<0.001) deterring of IR-induced NFκB promoter activation almost to the basal levels (Figure 1B). Inhibition of NFκB promoter activation 24h post-IR portrays the potential of disrupting the second-signaling feedback.

Moreover, to delineate that EF24 regulates the IR-associated incessant IκBα phosphorylation, SH-SY5Y, IMR-32, SK–PNDW and MC-IXC cells exposed to IR with/without EF24 (200nM for 3h) were examined after 12h through 72h. Recently, we showed that at least in human NB cells, IR persistently induced IκBα phosphorylation for up to 3 days [13]. In this study, treating NB cells with EF24 significantly suppressed this IR-induced IκBα phosphorylation (Figure 1C). EF24-associated suppression of IR-induced IκBα phosphorylation was observed as early as 12h post-IR and remained consistent for up to 72h in all cell lines investigated (Figure 1C). More importantly, immunoblotting analysis revealed that EF24 treatment resulted in the significant inhibition of p50/p65 overexpression induced IκBα phosphorylation in SHSY5Y and SK–PNDW cells. Together, these data suggests that EF24 attenuates IR-triggered NFκB-TNFα PFC-dependent NFκB maintenance through consistent blocking of post-translational modification of IκBα in this setting.

### EF24 regulates IR-induced TNFα transactivation and intercellular secretion

To determine whether IR-induced NFκB muting efficacy of EF24 essentially translates to the disruption of the TNFα-dependent second signaling feedback, we investigate the potential of EF24 in mitigating IR-induced NFκB-dependent TNFα transactivation and secreted TNFα mediated feedback [26]. SH-SY5Y and IMR-32 cells either exposed to mock-IR, 2Gy with or without EF24 (200nM) treatment were examined after 1h through 24h for alterations in *TNFα* mRNA levels. Likewise, *TNFα* mRNA modulations were examined after muting either IR-induced NFκB (RelA siRNA) or blocking auto/paracrine TNF ligand/receptor binding (TNFR1 Ab). The ^ΔΔ^ *C*t values were calculated by normalizing the gene expression levels to β-actin, and the relative expression level was expressed as a fold change over mock-irradiated (untreated) control. IR significantly (P<0.001) induced *TNFα* transactivation for up to 24h in both cell lines. Consequently, when IR-induced NFκB was muted, we observed a significant (P<0.001) suppression in IR-induced *TNFα* transactivation (Figure 1D& 1E). Similarly, when we block the binding back of secreted TNFα to its receptor, second-signaling-dependent *TNFα* transactivation is completely muted. More importantly, pretreatment with EF24 profoundly inhibited IR-induced TNFα transactivation as early as 1h post-IR. Interestingly, this EF24-associated significant (P<0.001) inhibition of IR-induced TNFα in surviving NB cells remained consistent at least after 24h in SH-SY5Y (Figure 1D) and IMR-32 (Figure 1E) cells.

In addition, 9Kd cutoff concentrated medium recovered after 24h through 72h from SH-SY5Y and IMR-32 cells either exposed to 2Gy with/without EF24, RelA-siRNA transfected with IR-exposure or treated with TNFR1Ab and exposed to IR were examined for secreted TNFα. IR significantly increased secreted TNFα after 24h and remained consistent up to 72h (Figure 1F& G). Muting IR-induced NFκB significantly reduced TNFα secretion validating that NFκB maintenance mediates TNFα. Similarly, hindering secreted TNFα binding-back with TNFR1-Ab profoundly blocked the subsequent TNFα secretion. Importantly, EF24 treatment showed a significant and consistent (for at least 3 days) repression in IR-induced TNFα secretion (Figure 1F& G) demonstrating its influential role in disrupting IR-induced NFκB-TNFα-NFκB feedback cycle.

### EF24 impedes IR-induced NFκB-dependent survival signaling

**Figure 2:**
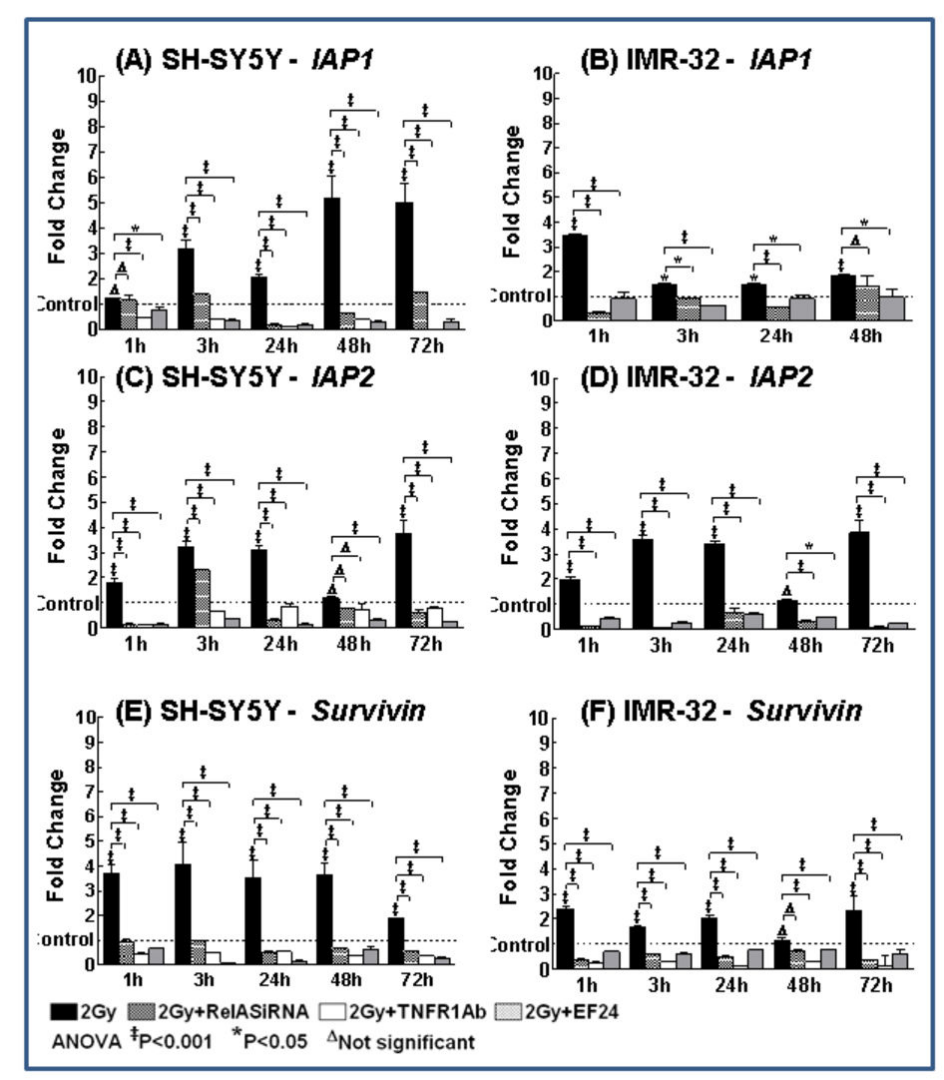
EF24 inhibits radiation-induced transactivation of NFκB downstream pro-survival targets in human neuroblastoma cells. Histograms showing *cIAP1, cIAP2* and *Survivin* transactivation (QPCR analysis) in SH-SY5Y and IMR-32 cells exposed to 2Gy with or without EF24 treatment, transfected with RelA siRNA or treated with TNFR1 Ab and exposed to 2Gy. The ΔΔ^ct^ values were calculated by normalizing the gene expression levels to internal housekeeping gene (*β-actin*), compared between groups, and the relative expression level was expressed as a fold change over mock-IR cells. Groups were compared using ANOVA with Tukey’s Post-hoc correction.

We have shown that IR-induced PFC-dependent NFκB-mediates survival advantage after RT [13] and, consequently, herein, we investigated the potential of EF24 in the regulation of IR-induced NFκB-mediated *IAP1, IAP2, Survivin* and subsequent survival advantage. NB cells either exposed to 2Gy, RelA-siRNA transfected with 2Gy exposure or treated with TNFR1Ab and exposed to IR were analyzed for *IAP1, IAP2* and *Survivin* transactivation. The ^ΔΔ^ *C*t values were calculated by normalizing the gene expression levels to β-actin, and the relative expression level was expressed as a fold change over mock-irradiated (untreated) control. IR profoundly induced *IAP1, IAP2* and *Survivin* mRNA levels as early as 1h and remained elevated at least up to 72h in both IMR-32 and SH-SY5Y cells (Figure 2A–F). Muting NFκB completely compromised IR-induced *IAP1, IAP2* and *Survivin* after 1h through 3 days (Figure 2). Also, blocking binding-back of TNFα markedly introverted IR-induced *IAP1, IAP2* and *Survivin* at time-points investigated (Figure 2A,C,E,F). Armed with the fact that IR-triggered PFC-dependent maintenance of NFκB mediates the regulation of survival signaling [13], we elucidated the efficacy of EF24 in attenuating survival signaling by selectively disrupting PFC. EF24 significantly inhibited the IR-induced NFκB-dependent *IAP1, IAP2* and *Survivin* transactivation as early as 1h in both cell lines (Figure 2). Interestingly, consistent with EF24-mediated inhibition TNFα and NFκB, we observed a sustained inhibition of pro-survival molecules demonstrating the effect of EF24 in mitigating IR-induced PFC-dependent NFκB-mediated survival signaling.

**Figure 3:**
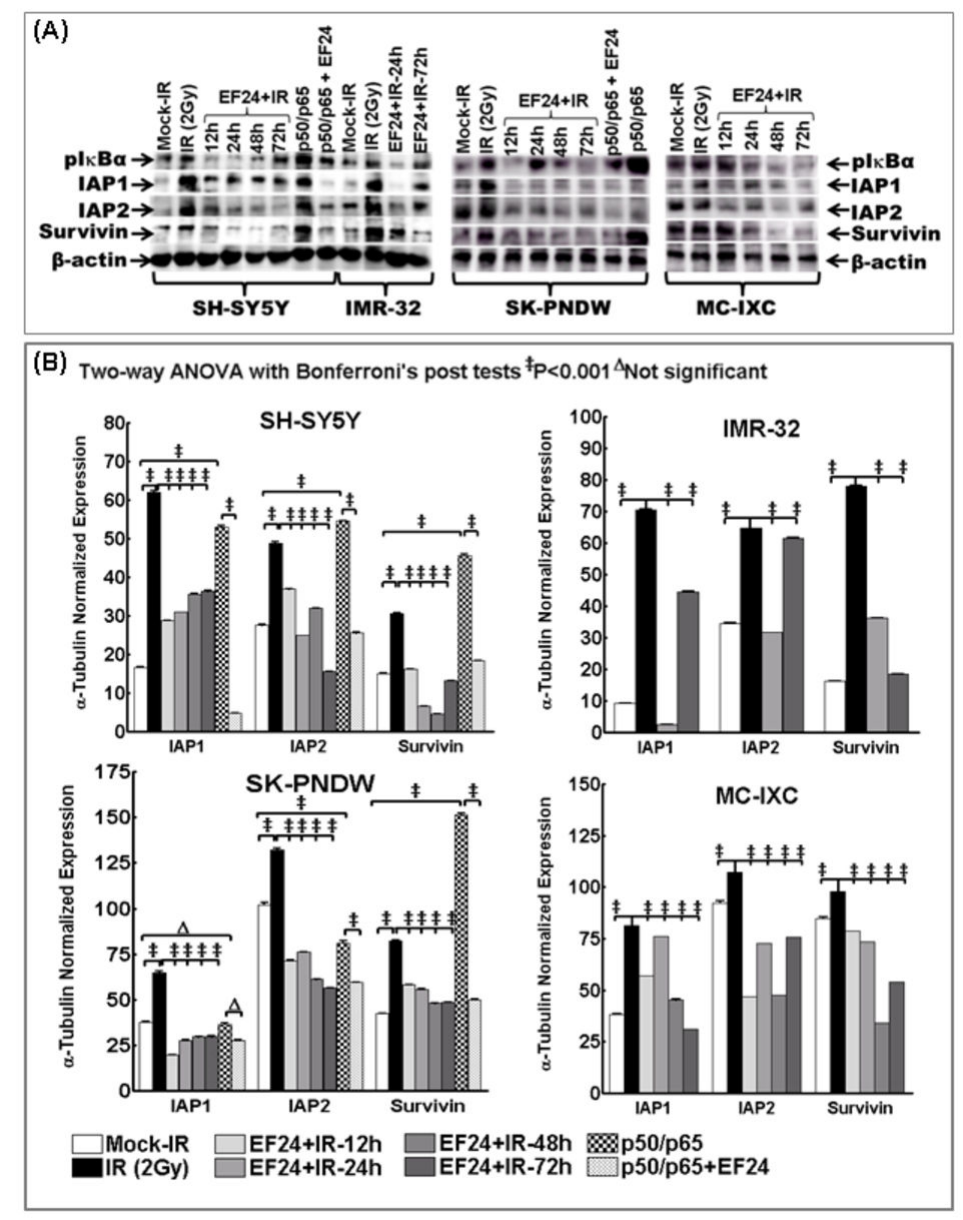
EF24 regulates radiation-induced NFκB downstream pro-survival proteins in human neuroblastoma cells. (**A**) Immunoblots showing alterations in pIκBα, IAP1, IAP2 and Survivin protein expression in SH-SY5Y, IMR-32, SK–PN–DW and MC-IXC cells either mock-irradiated, exposed to 2Gy and harvested after 24h, treated with EF24 for 3h followed by 2Gy exposure and harvested after 1, 3, 12, 24, 48 and 72h, transfected with p50/p65 for 24h or transfected with p50/p65 for 24h and treated with EF24 for additional 24h. (**B**) Semi-quantitative densitometry of immunoblots using Quantity One 1D image analysis Version 4.6.5 (Biorad) showing α-tubulin intensity normalized expression of IAP1, IAP2 and Survivin in SH-SY5Y, IMR-32, SK–PN–DW and MC-IXC cells. Groups were compared using Two-way ANOVA with Bonferroni’s Post-hoc correction.

Immunoblotting confirmed the efficacy of EF24 in targeting NFκB-dependent survival signaling in human NB cells (Figure 3A). EF24 significantly (P<0.001) repressed IR-induced IAP1, IAP2 and Survivin in SH-SY5Y, IMR-32, SK–PNDW and MC-IXC cells (Figure 3A& B). EF24 induced inhibition of the IAPs remained consistent for at least 72h. Further, activating NFκB resulted in a significant (P<0.001) upregulation of IAP1, IAP2 and Survivin, signifying the influential role of NFκB. Notably, treating NFκB-overexpressed cells with EF24 exhibited complete inhibition of survival molecules (Figure 3). Together, the IAPs transactivation/translation data demonstrates that IR-induced sustained NFκB is required for the consistent influence of survival signaling and, EF24 potentially reverted this IR-induced NFκB-TNFα cross-signaling mediated NFκB dependent regulation of IAPs. NFκB muting/overexpression and TNFα ligand/receptor blocking studies validate the signaling involved in EF24-mediated inhibition

### EF24 thwarts IR-induced NFκB-regulated NB survival advantage

**Figure 4:**
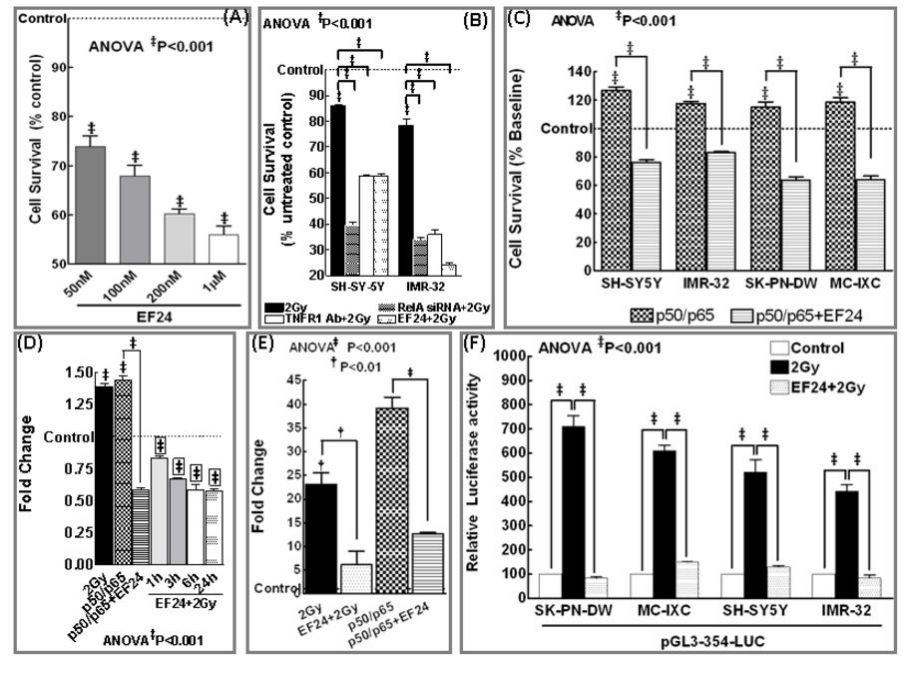
EF24 regulates radiation-induced NFκB dependent hTERT transactivation, transcription and confers radiation induced cell killing. (**A**) MTT analysis showing survival response in human SH-SY5Y cells exposed to EF24 (50, 100, 200nM, 1μM). Induced inhibition of cell survival was compared to mock-IR control. (**B**) Histograms of MTT analysis showing cell survival response in SH-SY5Y and IMR-32 cells either exposed to IR (2Gy) with or without EF24, transfected with RelA siRNA and exposed to IR or treated with TNFR1 Ab and exposed to IR. (**C**) MTT analysis showing inhibition of NFκB dependent survival response in NFκB (p50/p65) overexpressed human SH-SY5Y, IMR-32, SK–PN–DW and MC-IXC cells with EF24 treatment. Groups were compared using ANOVA with Tukey’s Post-hoc correction. Histograms showing *hTERT* mRNA expression assessed by QPCR analysis in (**D**) SH-5Y5Y and (**E**) IMR-32 cells mock-irradiated, exposed to 2Gy, treated with EF24 for 3h followed by 2Gy exposure and harvested after 1, 3, 6 and 24h, transfected with p50/p65 with or without EF24 treatment. The ΔΔ^ct^ values were calculated by normalizing the gene expression levels to internal housekeeping gene (*β-actin*), compared between groups, and the relative expression level was expressed as a fold change over mock-IR cells. ANOVA with Tukey’s post hoc correction was used to compare between groups. (**F**) Luciferase reporter assay: SH-SY5Y, IMR-32, SK–PN–DW and MC-IXC cells transfected with pGL3-354-Luc construct and either mock irradiated, exposed to 2Gy, treated with EF24 and exposed to 2Gy were harvested at 24h post-IR and analyzed by luciferase assay. Groups were compared using ANOVA with Tukey’s Post-hoc correction. Data shown represent the mean and SD of three independent experiments.

Further to substantiate the potential of EF24, we investigated the functional response, inhibition of cell survival in this setting. Stand-alone EF24 (50nM-1µM) treatment resulted in a significant (P<0.001) and dose-dependent reduction in SH-SY5Y cell survival (Figure 4A). Evidently, while, IR (P<0.001) reduced the cell survival, disrupting IR-induced feedback by blocking TNFα receptor or silencing NFκB profoundly conferred IR-inhibited cell survival (Figure 4B). More importantly, EF24 further potentiated (P<0.001) IR-inhibited cell survival in both NB cell-lines (Figure 4B). Conversely, NFκB overexpression in SH-SY5Y, IMR-32, SK–PN–DW and MC-IXC cells significantly (P<0.001) induced cell survival (Figure 4C). EF24 completely (P<0.001) suppressed NFκB-induced cell survival in all these cell lines. Altogether, these data demonstrate that, EF24 potentially inhibits cell survival in a dose dependent manner; EF24 significantly radiosensitizes these cells by conferring IR-induced cell death and; that EF24-induced cell killing involves regulation of IR-induced persistent NFκB.

### EF24 inhibits IR-induced NFκB-dependent TERT and TA

**Figure 5:**
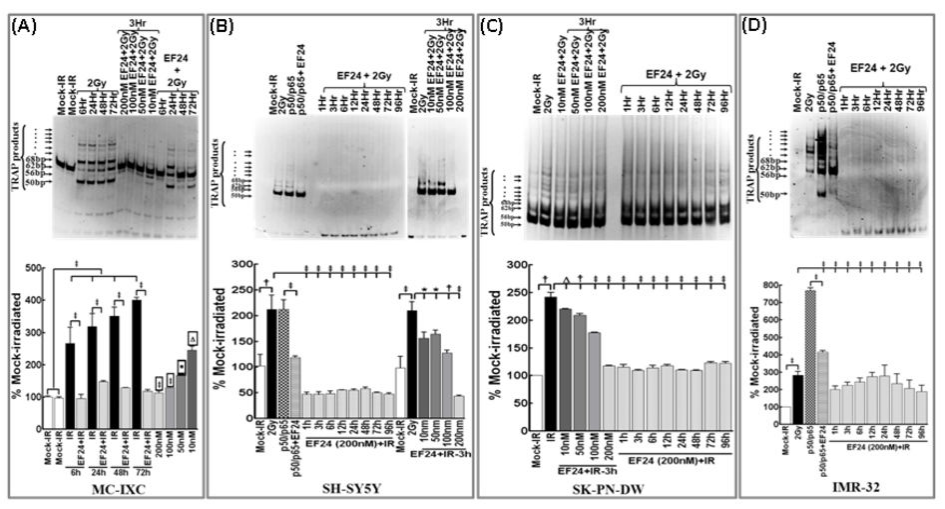
EF24 regulates radiation-induced telomerase activation in human neuroblastoma cells. Representative gels and corresponding densitometry analysis showing telomerase activity in (**A**) MC-IXC, (**B**) SH-SY5Y, (**C**) SK–PN–DW and (**D**) IMR-32 cells either mock-irradiated; exposed to 2Gy and harvested after 6, 24, 48 and 72h; treated with 10, 50, 100 and 200nM EF24 for 3h followed by IR exposure and harvested after 3h, treated with 200nM EF24 for 34 followed by IR exposure and harvested after 1, 3, 6, 12, 24, 48, 72 and 96h; transfected with p50/p65 for 24h or transfected with p50/p65 for 24h and treated with EF24 for additional 24h. Densitometry analysis with automatic band detection (ImageQuant TL, Amersham Biosciences) showed significant inhibition of either 2Gy- or p50/p65-induced telomerase activity with EF24.

SH-SY5Y and IMR-32 cells either exposed to IR with/without EF24 or NFκB overexpressed and treated with/without EF24 were examined for TERT mRNA alterations. The ^ΔΔ^ *C*t values were calculated by normalizing the gene expression levels to β-actin, and the relative expression level was expressed as a fold change over mock-irradiated (untreated) control. IR significantly induced *TERT* transactivation in SH-SY5Y (Figure 4D) and IMR-32 (Figure 4E) cells. Interestingly, we observed a robust increase in TERT transactivation in IMR-32 cells. EF24 treatment profoundly and consistently suppressed this IR-induced *TERT* transactivation in both cell-lines. Further, NFκB overexpression significantly (P<0.001) increased *TERT* mRNA in both cell lines (Figure 4E). EF24 completely reverted NFκB-induced *TERT* transcription. Further, to investigate the efficacy of EF24 in reverting IR-induced NFκB-dependent TERT transcription, SH-SY5Y, IMR-32, SK–PN–DW and MC-IXC cells transfected with a pGL3-354-Luc were exposed to IR with/without EF24 treatment and then analyzed for luciferase activity. IR exposure substantially increased (P<0.001) luciferase activity, demonstrating a NFκB-dependent functional transcription of TERT in these cells (Figure 4F). We have shown that cells transfected with same TERT promoter containing plasmid but lacking NFκB binding sites did not show any promoter activation after IR [16]. Interestingly, this IR-induced TERT promoter activation was profoundly (P<0.001) suppressed with EF24 which signifies the potential efficacy of EF24 in attenuating the IR-induced NFκB-dependent functional TERT transcription (Figure 4F). In addition, TRAP analysis showed that IR significantly (P<0.001) induced TA (Figure 5). More importantly, EF24 treatment resulted in a significant (P<0.001) and dose dependent inhibition of IR-induced TA in MC-IXC, SH-SY5Y and SKPN-DW (Figure 5A–C) cells. TA kinetics after IR with or without EF24 revealed that IR significantly (P<0.001) induced TA in MC-IXC cells at 6h post-IR and this increase remained sustained at least after 72h (Figure 5A). Conversely, EF24 treatment showed a complete and persistent abrogation of TA consistently 1h through 96h in all four cell-lines investigated (Figure 5). In addition, NFκB overexpressed SH-SY5Y and IMR-32 cells revealed a robust induction (P<0.001) of TA. Consequently, EF24 treatment completely silenced NFκB-induced TA (Figure 5B& D). Taken together, these data demonstrates that EF24 regulates IR-triggered PFC-induced NFκB maintenance-dependent TERT-mediated TA in human NB.

### EF24 mitigates IR-induced NFκB-dependent NB clonal expansion

**Figure 6:**
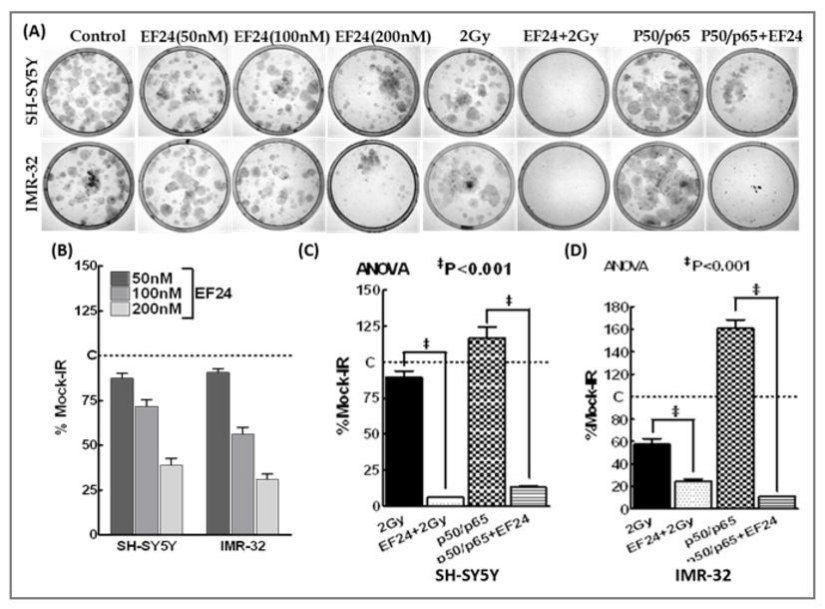
EF24 inhibits radiation-induced NFκB dependent initiation of clonal expansion in human neuroblastoma cells. (**A**) Representative images showing clonogenic activity of SH-SY5Y and IMR-32 cells either mock irradiated, treated with increasing (50, 100 and 200nM) concentrations of EF24 or exposed to 2Gy with or without EF24 (200nM) treatment, transfected with p50/p65 with or without EF24 treatment. The colonies were fixed, stained with 0.5% crystal violet, and imaged. (**B**) Histograms showing dose dependent inhibition of clonal expansion in Ef24 treated SH-SY5Y and IMR-32 cells. Histograms showing clonogenic capacity of (**C**) SH-SY5Y and (**D**) IMR-32 cells exposed to IR, treated with EF24 and exposed to IR, transfected with p50/p65 and treated with or without EF24. The colonies were counted using computed colony counting (Image Quant). Groups were compared using ANOVA with Tukey’s Post-hoc correction.

In this study, in order to assess the efficacy of EF24 in inhibiting this IR-induced PFC dependent NFκB-mediated clonal expansion, we examined the induced modulations in clonogenic activity (Figure 6A). First to determine the potential of EF24 as stand-alone compound in this setting, SH-SY-5Y and IMR-32 cells exposed to increasing concentrations of EF24 (50, 100 and 200nM) were examined for the inhibition of clonal expansion. EF24 significantly inhibited NB cell clonal expansion with as low as 50nM. Increasing concentrations of EF24 revealed a dose dependent decrease in clonal expansion in these cells (Figure 6B). Next to determine the radiosensitizing potential of EF24, NB cells pretreated with EF24 (200nM) were exposed to IR. IR exposure notably inhibited clonal expansion in both cell lines. However, this IR-inhibited clonal expansion were further (P<0.001) suppressed with EF24 treatment (Figure 6C-D). Finally to determine whether EF24 targets NFκB dependent clonal expansion, NFκB activated cells treated with or without EF24 were examined. NFκB activation significantly increase NB cell clonal expansion. Conversely, EF24 completely (P<0.001) introverted NFκB activation-induced clonal expansion in NB cells (Figure 6C-D). Together, these data delineates that EF24 could completely suppress the IR-induced NFκB-dependent clonal expansion in NB cells.

### Intra-tumoral EF24 regress NB and alleviates IR-induced NFκB and TNFα

**Figure 7:**
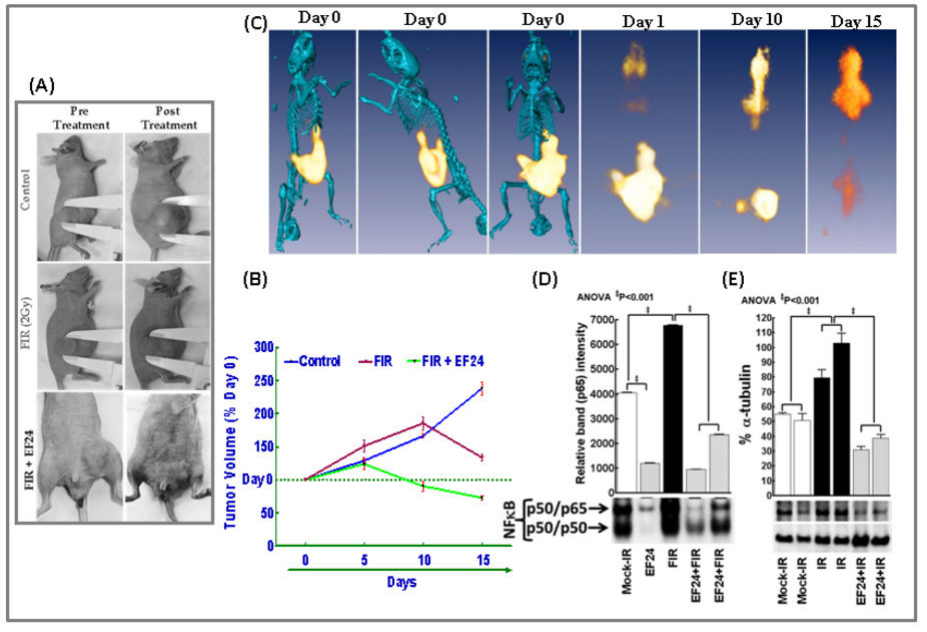
Intra-tumoral EF24 regress tumor growth in NB xenograft mice. (**A**) Representative photographs showing variations in tumor size prior to and after treatment in NB xenograft mice. The mice were either mock-irradiated, exposed to FIR (2Gy/D for 5D/Wk to a total dose of 20Gy) or treated with daily dose of intra-tumoral EF24 (200µg/Kg) in conjunction with FIR. (**B**) Line plot of NB tumor volume depicting incessant NB progression in mock-IR animals and significant NB regression after EF24 administration in conjunction with FIR exposure as opposed to FIR exposure alone. (**C**) FDG-PET-CT over-imposed images showing intra-tumoral EF24 administration associated tumor regression in athymic nude mice bearing NB xenograft. (**D**) NFκB DNA-binding activity in NB xenografts exposed to FIR, treated with intra-tumoral EF24 with or without FIR exposure. The nuclear extracts from treated xenografts were analyzed by EMSA using γ-^32^p[ATP] labeled NFκB-specific probe. Semi-quantitative densitometry of autorads using Quantity One 1D image analysis (Biorad) showed complete inhibition of FIR-induced NFκBDNA-binding activity in human NB xenografts treated with EF24.Groups were compared using ANOVA with Tukey’s Post-hoc correction. (**E**) Representative immunoblot and corresponding densitometry showing TNFα levels in mock-irradiated and irradiated (with or without intra-tumoral EF24) NB xenografts. Semi-quantitative densitometry of immunoblots using Quantity One 1D image analysis (Biorad) showing α-tubulin intensity normalized expression of TNFα. Groups were compared using ANOVA with Tukey’s Post-hoc correction.

Establishing the practical feasibility, possibility of tumor-targeted delivery and ascertaining the clinically translatable effectiveness of EF24 in preclinical model is imperative to successfully put this drug to use in the cure of NB. Accordingly, we tested whether intra-tumoral delivery of EF24 (200µg/Kg) impedes NB growth in xenograft model (Figure 7A). Tumor growth (progression/regression) was determined by standard tumor volume measurements (Figure 7B) and validated with FDG-PET-CT imaging (Figure 7C). The control xenografts demonstrated a steady pace tumor growth (238.3±10.29% over day 0) without any signs of regression until the end of experimental timeline. Though, FIR (2Gy/Dx5D/Wk -total dose of 20Gy) exposure showed an initial increase (185.6±9.55% on Day10), we observed a tumor regression pattern there after (133.79±4.4% on day 15). Conversely, animals treated with intra-tumoral EF24 prior to FIR demonstrated a profound reduction in tumor volume by 73.10±2.9% (Figure 7A& B). To substantiate these results, we analyzed the metabolic activity of NB xenograft in animals following mock-IR and FIR exposure with or without intra-tumoral EF24. The NB xenografted mice were imaged before the start of experiment (day 0) as well as at Day1, Day 10 and at the end of the experiment on Day 15. In corroboration with the tumor volume data, FDG-PET image analysis revealed a massive increase in metabolic activity in mock-IR NB xenograft from day 0 until the end of experiment. However, intra-tumoral EF24 controlled NB growth and metabolic activity which confirms the potential efficacy of EF24 in this setting (Figure 7C). EMSA analysis on the NB xenografts showed that, while FIR exposure robustly (P<0.001) induced NFκB-DNA binding activity, intra-tumoral EF24 in conjunction with FIR showed a complete suppression in this IR-induced NFκB (Figure 7D). Also, immunoblotting revealed a significant (P<0.001) increase of TNFα in NB xenografts exposed to IR. However, we observed a total alleviation of this IR-induced TNFα (even to a lesser degree than the basal level) with intra-tumoral EF24 (Figure 7E).

**Figure 8:**
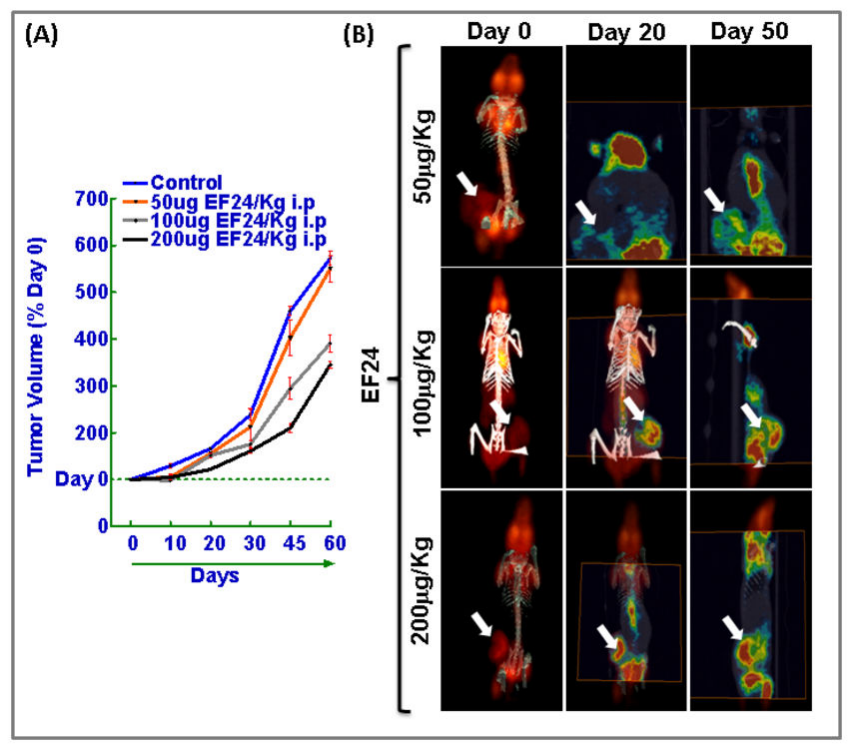
Dose-dependent regression of neuroblastoma by EF24 treatment. (**A**) Line plot of NB tumor volume depicting relentless NB progression in mock-IR animals and significant NB regression after increasing concentrations (50, 100 or 200mg) of EF24 (*i.p.* daily-dose) administration. The mice were either mock-irradiated or treated with daily dose of intra-peritoneal EF24 (200µg/Kg). (**B**) FDG-PET-CT over-imposed images showing daily dose of intra-peritoneal EF24 associated tumor regression in athymic nude mice bearing NB xenograft.

Further to establish the dose response curve, animals bearing NB xenografts were exposed to daily dose (*i.p.*) *of* increasing concentrations (50, 100 or 200mg/Kg) of EF24 (Figure 8). The untreated xenografts revealed a steady pace tumor growth (572.7±15.0% over day 0) without any regression until the end of experimental timeline. Evidently, daily dose of *i.p.* EF24 resulted in a dose dependent reduction in tumor volume by 550.31±28.19%; 390.9±19.0% and 345.5±8.03% after 50, 100 and 200mg/Kg of EF24 respectively (Figure 8A). To validate our findings, FDG-PET image analysis were performed in these animals on Day 0, Day 20 and at Day 50 (Figure 8B). In corroboration with the tumor volume data, FDG-PET image analysis revealed a massive increase in metabolic activity in untreated control NB xenograft from day 0 until day 50. Conversely, daily dose of intra-peritoneal EF24 induced a dose-dependent regulation of NB growth and metabolic activity. Notably we found a profound decrease in tumor metabolic activity in animals that received 200mg/Kg EF24 (Figure 8B).

**Figure 9:**
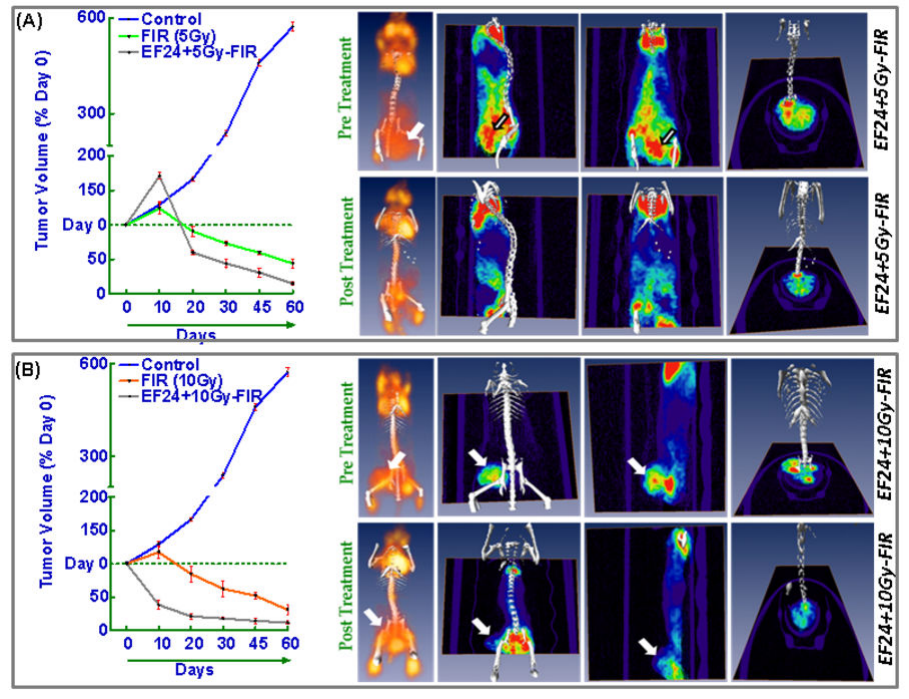
EF24 in conjunction with hypo-fractionated radiation regress neuroblastoma. Tumor volume line plots and FDG-PET-CT over-imposed images of NB xenografts of animals treated with daily dose (*i.p.*) EF24 (200mg/Kg) in conjunction with or without (**A**) 5Gy-FIR and (**B**) 10Gy-FIR. NB xenografts were selectively irradiated with 5Gy-FIR of 10Gy-FIR to a total dose of 75 and 70Gy respectively, with a daily dose of *i.p.* EF24. Hypo-fractionated IR induced NB regression was significantly conferred in the presence of EF24. FDG-PET imaging was performed on Day 0 and at Day 50.

Next, we investigated the radiosensitizing potential of EF24 in hypo-fractionated radiation dose regimens. For this NB xenografts were selectively irradiated with 5Gy-FIR of 10Gy-FIR to a total dose of 75 and 70Gy respectively, with a daily dose of *i.p.* EF24 (Figure 9). Compared to the mock-irradiated xenograft progression (572.7±15.0% over day 0), hypo-fractionated IR significantly induced NB regression (5Gy-FIR, 44.1±6.41%; 10Gy-FIR, 30.7±7.00%). However, this hypo-fractionated IR induced NB regression was significantly conferred (5Gy-FIR+EF24, 14.9±2.84%; 10Gy-FIR+EF24, 11.7±2.29%) in the presence of EF24 (Figure 9A& B). FDG-PET imaging was performed in these animals on Day 0 and at Day 50 (Figure 9). In corroboration with the tumor volume data, FDG-PET image analysis revealed a massive increase in metabolic activity in untreated control NB xenograft from day 0 until day 50. Conversely, daily dose of intra-peritoneal EF24 in conjuction with either 5Gy-FIR or 10Gy-FIR induced a significant inhibition of NB growth and metabolic activity. Notably we found a profound decrease in tumor metabolic activity in animals that received 200mg/Kg EF24 coupled with 10Gy-FIR (Figure 8B).

## Discussion

The cure of NB, the childhood extra-cranial solid tumor with a broad spectrum of clinical behavior presents imminent challenge due to its high propensity to spread. As discussed earlier, the cure rate is only 10% in children with stage 4S NB who show systemic recurrence. As RT is widely used in the treatment of NB in combination with chemotherapy, surgery, immunotherapy and/or stem cell therapy, any means of potentiating IR-induced cell killing and mitigating radioresistance in surviving tumor cells would highly benefit these children. To that end, NB treatments targeting key signaling pathways operating in response to treatment modalities, here in this case, RT, to limit both the local relapse and progression of distant metastasis is warranted. Recently, we delineated the “functionally-specific” canonical and second signaling orchestration of transcriptional switch, NFκB in NB after clinical RT and its downstream regulatory mechanisms influencing survival advantage and clonal expansion [13,16]. Evidence from our report [14,15,31] and others [32] have dictated that, developing pharmacologically safe antagonists of transcription factors, here in this case, NFκB may prove beneficial in childhood cancer prevention and treatment. In this study, for the first time, we identified the potential of synthetic monoketone, EF24, an analog of curcumin with enhanced physicochemical properties in targeting RT-associated NFκB orchestration and validated its efficacy in mitigating NB cell survival advantage and clonal expansion. Inimitably, this study identified the potential of EF24 in selectively targeting IR-induced NFκB-TNFα cross-signaling and thereby crumples maintenance of NFκB.

Several classes of NFκB inhibitors are currently being tested in combination with RT including IKK inhibitors [33], inhibitory peptides [34], antisense RNA [35,36], proteasome inhibitors [37,38] and dietary agents that can block at various steps leading to NFκB activation and sensitize the tumor cells to the beneficial effects of radiation. Incidentally, radiosensitization of tumors have been reported with a number of plant polyphenols and an impressive number of clinical trials are currently ongoing to test the efficacy of drugs that could specifically inhibit NFκB (www.clincaltrials.gov). In this regard, we recently demonstrated the potential efficacy of dietary polyphenol, curcumin to inhibit IR-induced persistent NFκB activation and the IR-induced NFκB-dependent *TERT* transactivation, TA and clonal expansion in human NB cells [16]. However, the scientific and clinical research demonstrated that the targeted delivery, efficacy and safety of curcumin in NB, for that matter, in any tumor system have not kept pace with the disease prevailing patterns. Consequently, this study investigated the efficacy of EF24, a more potent synthetic analog of curcumin, to inhibit IR-induced persistent NFκB activation and the resultant survival advantage and clonal expansion in NB cells. Results of the present study, for the first time comprehensively demonstrates that this novel monoketone (1) selectively targets RT-induced NFκB; (2) disrupts IR-induced NFκB-TNFα-NFκB feedback cycle and abrogates its associated maintenance of downstream pro-survival targets and NB cell survival as such; (4) attenuates NFκB-mediated TERT transcription, transactivation; (5) mitigates NFκB-dependent telomerase activation and clonal expansion; and (6) completely regressed NB in an *in vivo* setting and regulates IR-induced NFκB and TNFα in human NB xenograft.

EF24 has been shown to induce apoptosis in cancer cells and inhibit the growth of human breast tumors in a mouse xenograft model with relatively low toxicity and at a dose much less than that of curcumin [19,20]. EF24 has been shown to induce cell cycle arrest and apoptosis in many cancer cell lines, with potency much higher than that of curcumin. More recently, Kasinski et al., reported that EF24 exhibits IC50 values of 10 to 20 times lower than that of curcumin in a panel of non-small cell lung cancer cells with different genetic background as well as in ovarian, cervical, breast and prostate cancer cells [39]. Also, studies with various cancer cells have suggested that EF24 impairs cell growth by inducing G2/M arrest followed by induction of apoptosis, which is accompanied by caspase-3 activation, phosphatidylserine externalization and an increased number of cells with sub-G1 DNA content [20]. However, the cell signaling pathways that mediate the EF24 effect are poorly understood. To that end, Kasinski and colleagues causally correlated the amount of EF24 required for the suppression of lung cancer A549 cell growth to its ability to prevent the nuclear translocation of p65 subunit of NFκB, to block IκB phosphorylation and its subsequent degradation, and to inhibit catalytic activity of the IKK protein complex [39]. In this context, results of the current study clearly elucidated the potential of EF24 in suppressing IR-induced NFκB DNA binding activity and functional NFκB promoter activation in all NB cell lines investigated. More importantly, with the inhibition of NFκB, EF24 also suppressed the IR-induced sustained TNFα transactivation and intercellular secretion. Thus direct targeting of IR-induced NFκB-TNFα-NFκB feedback cycle may explain the potential of EF24 in inhibiting persistent maintenance of NFκB in these cells. Although EF24 has been shown to inhibit IKKβ kinase activity [24], this study has precisely shown for the first time that, EF24 specifically intervenes IR-induced initiation of a NFκB-TNFα feedback cycle and blocks the PFC-dependent persistent NFκB activation with no recovery from this induced inhibition.

Further, to substantiate the benefit of EF24 in mitigating IR-induced PFC-dependent NFκB in NB cell survival as such, we performed cytotoxic assays with EF24 as a stand-alone compound, EF24 in combination with IR, and EF24 with or without manipulating NFκB activation. Our results clearly demonstrated a significant correlation of cell killing to the amount of EF24. New to science, our studies demonstrated that EF24 profoundly conferred IR-induced cell killing, survival signaling and more importantly, the results depict that EF24 targets IR-induced NFκB for this causal effect. To that note, studies have shown that, EF24 has potent anti-proliferative activity against a number of cancer cell lines including colon [21], breast [22], and ovarian [23]. Synthetic chemical analogues to molecularly targeted chemotherapeutic drugs and chemopreventive photochemical confound a myriad of molecular events in host and tumor tissues. These events include the acquisition of self-sufficient growth signals, insensitivity to signals that usually inhibit proliferation, use of survival pathways to evade apoptosis, initiation of angiogenesis to ensure sufficient oxygen and nutrient supply, and attainment of the ability to invade and metastasize [40]. EF24 molecule interferes with the progression of cancer by disrupting many of the characteristic cancer-promoting events more effectively than curcumin. Our data imply that EF24 treatment leads to dysregulation of NFκB downstream pro-survival IAP1, IAP2 and Survivin in a NFκB-targeted manner. This was further supported through our observation that EF24 disrupts IR-induced NFκB-TNFα cross-signaling.

Several studies have demonstrated that NFκB regulates the transcriptional activation of hTERT [41]. Telomerase activity (TA) is found in 85-90% of all human cancers, but not in their adjacent normal cells [42,43]. TA has been widely studied as a biomarker for the diagnosis and prognosis of various adult and childhood neoplasms including NB and is targeted for the development of novel therapeutic agents [44,45]. Moreover, the expression of TERT (catalytic subunit of telomerase), correlates with telomerase activity [46] and upregulation of TERT expression plays a critical role in human carcinogenesis [47] suggesting that TERT is a good target for cancer therapy. In addition, in our recent study, we have clearly shown that IR-induced persistent NFκB activation mediates hTERT expression, transcription and activation of telomerase leading to subsequent cell survival after IR in NB cells [16]. Inhibition of hTERT expression results in loss of telomere and could limit the growth of cancer cells. Correspondingly, studies have demonstrated that antisense oligonucleotides against TERT resulted in inhibition of telomerase activity and induction of apoptosis in ovarian and prostate cancer cells [48]. To that note, our data strongly imply that EF24 exerts a profound inhibitory effect on IR-induced functional TERT promoter activation as well as the IR-induced NFκB-dependent persistent *TERT* transactivation and consequent telomerase activation in all NB cell lines studied. In addition, our results, for the first time, demonstrated that EF24 effectively targets IR-induced NFκB-dependent *TERT* transactivation, telomerase activation and subsequent clonal expansion in human NB cells. Increased expression of TERT and telomerase activation has been demonstrated to be directly related to maintenance of cellular replicative immortality which is crucial for tumor progression. In this regard, results of the current study have clearly shown that EF24 has an immense potential to greatly confer the IR-inhibited clonal expansion of NB cells. Notably, NFκB overexpression studies have precisely delineated that, this conferring effect of EF24 on NB cell clonal expansion is through the regulation of IR-induced NFκB-dependent hTERT transcription-telomerase activation.

In conclusion, this study comprehensively identifies the potential of novel synthetic monoketone, EF24 in surviving NB cells after a course of radiotherapy. Precisely, EF24 targets IR-induced NFκB-triggered NFκB-TNFα-NFκB cross signaling and thereby prevents sustained maintenance of NFκB in the surviving NB cells. This selective targeting efficiently allows EF24 to mitigate NFκB-dependent survival advantage and clonal expansion. However, translation of this response in real terms of NB relapse and progression need more direct clinically translatable *in vivo* studies. Experiments to delineate this EF24 effect and also to identify NB targeted EF24 deliverable modalities are currently underway in our lab using human NB xenograft mouse model.
